# User violence in mental health services: Adaptation of an instrument. Healthcare-workers’ Aggressive Behavior Scale-Users-Mental Health Version (HABS-U-MH)

**DOI:** 10.1371/journal.pone.0212742

**Published:** 2019-03-04

**Authors:** José Antonio Ruiz-Hernández, María Sánchez-Muñoz, José Antonio Jiménez-Barbero, David Pina López, Inmaculada Galían-Muñoz, Bartolomé Llor-Esteban, Laura Llor-Zaragoza

**Affiliations:** 1 Department of Psychiatry and Social Psychology, University of Murcia, Faculty of Psychology, Espinardo, Murcia, Spain; 2 Mental Health Center of Cartagena, Murcia, Spain; 3 Occupational Risk Prevention Service, Murcia Health Service, Murcia, Spain; 4 Department of Education, Catholic University San Antonio, Faculty of Social Sciences and Communication, Guadalupe, Murcia, Spain; Università degli Studi di Perugia, ITALY

## Abstract

**Background/Objetive:**

According to the World Health Organization, one out of every four violent workplace acts takes place in the health setting. The aims of the study are to adapt the *Healthcare-workers’ Aggressive Behavior Scale-Users (HABS-U)* to mental health professionals, to establish the frequency of exposure to hostile indicators and to determine which professional group is most exposed.

**Method:**

Study through qualitative and quantitative methodology in MH professionals of the Region of Murcia (Spain). In the qualitative phase, 12 in-depth interviews were conducted, and during the quantitative phase, the instrument was applied to 359 professionals of Mental Health Services (MHS).

**Results:**

Non-medical and nursing staff were found to be the professional group most exposed, as well as Brief Psychiatric Inpatient and Medium-Stay Inpatient Services.

**Conclusion:**

The resulting scale shows excellent psychometric properties. The distribution of user violence is not homogeneous among the different professional groups of MHS. The adaptation of the scale may be useful to detect user violence, as well as to evaluate the efficacy of intervention programs.

## Introduction

Violence has become a phenomenon of growing social concern due to its presence in various areas of society, including the workplace. In recent years, workplace violence in the health sector has increased exponentially [1, 2] Our study focuses on Type II violence, defined by the Occupational Safety and Health Administration [3] as that in which there is some kind of professional relationship between the aggressor and the worker.

Workplace violence has been defined as incidents in which personnel suffers abuse, sexual harassment, threats, or attacks in work-related circumstances, which explicitly or implicitly endanger their safety, well-being, or health. One of its classifications would be: hostile behavior such as psychological violence (verbal abuse, intimidation, harassment, and threats) and physical violence (use of force against persons or property, which produces material, physical, sexual, or psychological damage) [4–6].

Most studies in the Mental Health area have reported a predominance of non-physical violence in the health sector, although in some units, like Emergency and Psychiatry units, physical violence is also present, most frequently expressed in Psychiatric units through behaviors such as hitting objects and slamming doors, and shoving, shaking, or spitting [7–11]. The main patient factors most frequently associated with violent behavior in the Psychiatry units are being male, young, involuntary admittance, diagnosis of schizophrenia and substance abuse, among others [2, 12–13]. On another hand, Magnavita [14], in a study comparing various hospital services, points out that the mental health professional is 45 times more likely to suffer physical assault than other risk professions. In the same vein, a review of the literature by Piquero, Piquero, Craig, and Clipper [15], showed that between 14% and 61% of these workers had been victims of violence at the hands of their patients.

Regarding the professional group, nurses are pointed out in several studies as the most exposed to violence [16–19]. In this sense, Bowers *et al*. [20] found that virtually all mental health nurses have been assaulted at some point.

Cornaggia *et al*. [16] reported that much of nursing care focuses on relationships, and that therefore, the quality of the interactions with patients and with each other has strong implications for patients’ well-being and their propensity to violence.

High violence within Mental Health services, as well as the great variability of data due to the diverse methodologies, instruments and evaluation criteria used in the studies [11, 21] led us to study it through a specific scale. Therefore, this research is a continuation of previous studies examining user violence in the hospital setting [7, 20, 22] and in Primary Care [23] through the adaptation of the *Healthcare Workers’ Aggressive Behavior Scale-Users (HABS-U*) [24].

Serving as the theoretical and empirical reference framework, the objectives of this research were: to adapt the HABS-U to the Mental Health area, to determine the frequency of exposure to hostile indicators, and the most exposed professional group and type of service.

## Materials and methods

### Participants

The design of this study is analytical-descriptive, cross-sectional, and instrumental [25]. Participation was voluntary, and strict confidentiality and anonymity of the data collected was guaranteed. The study population involved Mental Health professionals from different health areas that depend on the Health Service of Murcia (southeast of Spain). The professionals were divided into four groups, according to the internal organization of Mental Health centers: psychiatrists, clinical psychologists, nursing staff, and non-medical personnel.

In the qualitative phase, we carried out a total of 12 in-depth interviews among the various professional categories. Concerning gender, 75% of the interviewees were women. The participants’ ages ranged between 25 and 55 years, with a mean age of 46.87 (*SD* = 9.92). Regarding profession, 62.5% were nursing staff (nurses and auxiliary nurses), 12.5% facultative staff (psychiatrists/psychologists), and the remaining 25% belonged to other categories (administrative, social workers,…).

Subsequently, in the quantitative phase, evaluation tools were applied to a sample of 359 professionals in the public network of Mental Health. In terms of the sociodemographic and work characteristics of the sample (Table 1), 26.3% were psychiatry professionals, 16.9% were clinical psychologists, 36.7% were nursing staff, and 20.1% were non-medical personnel. Participants’ mean age was 46.23 years (*SD* = 9.72), ranging between 25 and 65 years. The majority were women (70.7%), predominantly married or living with a partner (67.6%). In terms of the job profile, 32.6% of the professionals belonged to the Adult Unit, 5.8% to the Infant-Juvenile Unit, 10.4% to the Addictive Behavior Unit, 23.1% to Brief Psychiatric Inpatient Unit, 6.9% to Rehabilitation, and 7.5% to the Medium-stay Inpatient Unit.

**Table 1:** Sociodemographic and work characteristics of the sample.

Variable	*n*	*%*
**Age (years)**		
Younger than 35	46	13.3
36–45	110	31.9
46–55	113	32.8
56–65	76	22.0
Missing data	14	
**Sex**		
Male	104	29.3
Female	251	70.7
Missing data	4	
**Marital Status**		
Single	79	22.4
Common law partner or married	238	67.6
Divorced, separated, or widowed	35	9.9
Missing data	7	
**Sick leave** in the past 12 months?		
Yes	61	17.4
No	290	82.6
Missing data	8	
**Type of contract**		
Permanent	255	72.6
Temporary-substitution	96	27.4
Missing data	8	
**Professional group**		
Psychiatry	75	25.1
Clinical Psychology	45	15.1
Nursing	102	34.1
Non-medical staff	49	16.4
Missing data	28	
**Service**		
Adult Unit	112	32.6
Infant-Juvenile Unit	20	5.8
Addictive Behavior Unit	36	10.4
Rehabilitation Unit	24	6.9
Psychiatric Brief Inpatient Unit	80	23.1
Medium-Stay Inpatient Unit	26	7.5
Missing data	12	
**Job tenure (years)**		
<1	28	10.8
1–5	88	33.8
6–10	74	28.5
11–20	36	13.8
>20	34	13.1
Missing data	99	
**Professional tenure (years)**		
0–10	61	25.5
11–20	94	39.3
>20	84	35.1
Missing data	120	

### Instruments

- In order to assess user violence of low and medium intensity towards the professionals in the specialized care areas, we adapted the *Healthcare Aggressive Behavior Scale-Users* (HABS-U) [18]. It assesses the frequency of exposure on a 6-point Likert type scale ranging from 1 (never) to 6 (*every day*, *in the last year*) and consists of 10 items distributed in two factors: Non-physical violence (α = 0.85 and 36.4% explained variance) and Physical violence (α = 0.74 and 20.9% explained variance). In our study, it obtained an alpha of 0.877 for Non-physical violence, and of 0.841 for Physical violence.
-In addition to the HABS-U, socio-demographic and work variables (age, sex, marital and work status, type of service, seniority in the profession, job tenure, type of contract, shift, and overtime) were gathered in an ad-hoc questionnaire, as well as a validation scale protocol.
- *Maslach Burnout Inventory-General Survey (MBI-GS;* Schaufeli, Leiter, Maslach, & Jackson [26]; Spanish validated by version Gil-Monte [27]). This inventory contains 16 items with 3 dimensions (Emotional Exhaustion, Professional Efficacy, and Cynicism), which respondents rate on a 7-point Likert-type scale (0 = *never*; 6 = *always*). The reliability values in our sample (Cronbach’s alpha) were: 0.86 (Emotional exhaustion), 0.72 (Professional efficacy), and 0.66 (Cynicism).
- *General Health Questionnaire (GHQ-28;* Goldberg & Hillier [28]; Spanish adaptation by Lobo, Pérez-Echevarría, & Artal [29]). This 28-item inventory has 4 subscales: Somatic symptoms of psychological origin (Somatic GHQ), Anxiety/Insomnia (Anxiety GHQ), Social dysfunction (Dysfunction GHQ), and Depressive symptomatology (Depression GHQ). It is rated on a 4-point Likert scale ranging from 0 to 3, with higher scores indicating higher symptomatology intensity. In the present study, alphas of 0.85 (Somatic GHQ), 0.89 (Anxiety GHQ), 0.74 (Dysfunction GHQ), 0.84 (Depression GHQ) and 0.88 (Total scale GHQ) were obtained.
- *Overall Job Satisfaction (OJS;* Warr, Cook, & Wall [30]; Spanish adaptation by Pérez & Fidalgo [31]). This 15-item scale has 2 subscales: Intrinsic Satisfaction (7 items) and Extrinsic Satisfaction (8 items), and is rated on a 7-point Likert scale (0 = *very dissatisfied;* 6 = *very satisfied*)., In our sample, Cronbach alpha values of 0.88 (total scale) and of 0.86 and 0.71 (Intrinsic and Extrinsic Satisfaction; respectively) were obtained.
- *Jefferson Scale of Physician Empathy (JSPE;* Hojat, Mangione, Kane, & Gonnella [32]; Spanish adaptation by Alcorta-Garza, González Guerrero, Tavitas-Herrera, Rodríguez-Lara, & Hojat [33]). This 20-item scale has 3 subscales: Perspective Taking; Compassionate Care, and Standing in the Patient’s Shoes, and is rated on a 7-point Likert scale (1 = *completely disagree;* 7 = *completely agree*). In our sample, a Cronbach’s alpha of 0.52 was obtained for the total scale.

For the in-depth interviews, we used a script with the following questions: Which conflictive situations may occur between workers and users? Does this type of situation tend to occur frequently? How are hostile behaviors expressed (types of behavior: verbal, physical…)? Which behaviors are the most frequent? What acts do the patients perform? Do the same patients usually repeat the same behaviors?

### Procedure

Qualitative phase: in-depth interviews were performed to gather new data and complement the HABS-U items, using the described methodology. On the basis of the specialized bibliography, we developed a script adapted to mental health professionals. Key informants were contacted, performing interviews until information saturation was reached. The interviews were recorded for subsequent analysis.

Quantitative phase: all the centers of the Mental Health network of the Region of Murcia participated. We used randomized blocked sampling to select the sample. Meetings were held with the coordinators of each center, in which they were informed of the study, and the research protocol was randomly delivered to 50% of the staff of each center. Subsequently, we scheduled visits to the centers to clarify possible doubts and to collect the completed protocols. This study received the approval of the Committee of Research Ethics of the University of Murcia and the coordinators of the various participating health areas. The authors have no conflict of interest. This research was funded by the Faculty of Nursing, University of Murcia (17/2650). The funders had no role in study design, data collection and analysis, decision to publish, or preparation of the manuscript.

### Data analysis

Interviews were qualitatively analysed with theme analysis methodology [34]. According to its phases, the behavior categories (non-physical and physical violence) were identified from the interview transcripts. With this information, new items, which were reviewed and validated by a group of experts, were drafted by consensus, following previously established explicit criteria.

Using Hu and Bentler’s [35] methodology, which has been applied in similar studies [18, 24], we performed exploratory factor analysis (EFA) according to the following criteria: (a) factor selection was done through parallel analysis, following an iterative process; (b) each factor should explain at least 5% of the total variance; (c) items should load with at least 0.50 on their corresponding factor; (d) an item should not load with more than .30 on two factors; and (e) the items of each factor should have appropriate internal consistency (> 0.70). We used the least unweighted squares method with polychoric correlation matrices.

Subsequently, a confirmatory factor analysis (CFA) with Mplus was carried out using the weighted least squares means and variance-adjusted estimation (WLSMV) method for categorical data. From among the fit indices, the comparative fit index (CFI), Tucker-Lewis index (TLI), and the root mean square error of approximation (RMSEA) were selected. RMSEA < 0.05, and CFI and TLI ≥ 0.90 indicate a good fit of the model.

To assess the reliability of the resulting scale, we analyzed the internal consistency using Cronbach’s alpha for ordinal data. We examined its distribution through the means, standard deviation, skewness, and kurtosis, and lastly, confirmed external validity through the Pearson coefficient correlation between the scale and its factors with the validation scales.

We also performed a descriptive analysis of the sample and analyzed the annual prevalence of violent behavior through the indicators of the adapted scale, according to exposure frequency. Lastly, an *ANOVA* was used for multi-response variables along with the post hoc Tukey test to establish the differences between the different professional groups and the different mental health services.

Statistical analysis was performed with the SPSS statistical package version 22, Factor 10.3.01 and Mplus version 7.

## Results

Qualitative phase: using the qualitative analysis of the in-depth interviews, we created three categories as a function of user violence perceived by Mental Health workers. Below are some examples of literal narratives:

- Verbal violence: “They tend to raise their voices, they start screaming to tell you that they do not agree with some report you’ve made about them,” “They do not follow the rules well, they often protest about things they think are wrong…”
- Physical violence: “He stood up, grabbed my tie and choked me, and I fell down and fainted…”, “They hit the wall, or the closet where tobacco is kept, they may kick a chair…”
- Non-Verbal violence: “He looked at me defiantly”, “They interrupt the consultation; they open the door when you’re with someone else…”.

Four new items were drafted from the qualitative analysis of the interviews. The scale adapted to Mental Health (Healthcare-workers’ Aggressive Behavior Scale- Users- Mental Health Version [HABS-U-MH]) consisted of 10 items of the HABS-U plus four new items relating to aggressive behaviors or defiant attitudes.

Quantitative phase: the resulting scale was included in a protocol along with the rest of the study variables. A total of 400 questionnaires were distributed, and 359 correctly completed questionnaires were collected (89.75% response rate).

Using 50% of the sample (with simple random sampling, *n* = 179), an EFA was carried out. For this purpose, we analyzed the kurtosis and asymmetry of each item, finding that the physical violence items showed scores higher than 2, so we used the least unweighted squares method with polychoric correlation matrices [36, 37]. To assess the significance of the factor model, we used the Kaiser-Meyer-Olkin (KMO = 0.878) indexes and the Bartlett sphericity test (χ^2^ = 1141.4, *df* = 45, *p* ≤ .000).

The EFA yielded a scale consisting of 10 items. Through an iterative process [38], we eliminated three new items and one item from the original scale for not meeting the established criteria [18, 24]. Sixty-three percent of the variance of the scale was explained (α = 0.899). Its items are grouped into two factors: Factor I (Non-physical violence) with 6 items about users’ verbal and nonverbal violence, which explains 50.2% of the variance (α = 0.877); and Factor II (Physical violence), consisting of 4 items that account for 12.8% of the variance (α = 0.841). Table 2 shows the results obtained after examining the distribution of the scale, and Table 3 presents the EFA factor solutions.

**Table 2:** Descriptive statistics of the items.

	*M (SD)*	*RI T-c*	*α* *Without item*	*Skewness*	*Kurtosis*	*Communalities*	
						Initial	Extraction
**Non-physical Violence**							
Users question my decisions	3.05 (1.701)	0.733	0.867	0.419	-1.127	0.635	0.649
Users blame me for any trifle	2.53 (1.568)	0.758	0.863	0.821	-0.498	0.660	0.711
Users accuse me unfairly of not fulfilling my obligations, committing errors or complications	2.24 (1.463)	0.759	0.863	1.153	0.367	0.616	0.668
Users make ironic comments to me	2.33 (1.473)	0.668	0.877	1.001	-0.052	0.509	0.516
Users get angry with me because of assistential delay	2.68 (1.607)	0.615	0.886	0.715	-0.632	0.430	0.417
Users give me dirty or contemptuous looks	2.31 (1.492)	0.724	0.869	1.042	0.028	0.580	0.602
**Physical Violence**							
The users have even grasped me or touched me in a hostile way	1.61 (1.136)	0.769	0.861	2.123	3.975	0.691	0.696
Users have shoved me, shaken me, or spit at me	1.56 (1.059)	0.838	0.833	2.065	3.532	0.725	0.823
Users show their anger at me by breaking doors, windows, walls,…	1.51 (1.044)	0.719	0.878	2.218	4.418	0.593	0.607
Users have attacked me when I was trying to prevent their self-aggression*	1.44 (0.976)	0.736	0.873	2.474	5.574	0.556	0.610

* M = Media, RI T-c = Correlación ítem factor, α = Alpha Cronbach

**Table 3:** One-dimensional solution, two oblique factors and two orthogonal factors.

	1 Factor Solution	2 Orthogonal Factors Solution
χ^2^	χ^2^(35) = 414.748, *p* < .000	χ^2^(l26) = 88.130, *p* < .000
RMSEA	0.039	0.039
NNFI (Tucker & Lewis)	0.55	0.90
CFI	0.65	0.94
GFI	0.94	1
AGFI	0.92	0.99
Explained variance	4.69	
Factor I		50.2
Factor II		12.8
Reliability	0.903	
Factor I		0.868
Factor II		0.918

To analyze criterion validity, the correlations between the adapted scale and the validation scales employed were calculated (Table 4).

**Table 4:** Cronbach alphas and correlations between factors and psychosocial variables.

Variables	Cronbach’s alpha	HABS-U	Factor I: Non-physical violence	Factor II: Physical violence
***OJS***				
Total Satisfaction	0.883	- 0.348**	- 0.300**	- 0.329**
Extrinsic Satisfaction	0.710	- 0.326**	- 0.284**	- 0.304**
Intrinsic Satisfaction	0.860	- 0.332**	- 0.284**	- 0.318**
***JSPE***				
Perspective Taking	0.806	- 0.119*	- 0.080	- 0.160**
Compassionate Care	0.712	- 0.055	- 0.037	- 0.074
Standing in the Patient’s Shoes	0.426	- 0.158**	- 0.142**	- 0.144**
***MBI-GS***				
Emotional Exhaustion	0.864	0.156**	0.163**	0.092
Professional Efficacy	0.723	- 0.236**	- 0.226**	- 0.176**
Cynicism	0.661	0.116*	0.096	0.120*
***GHQ-28***				
GHQ Total	0.928	0.137*	0.132*	0.102
Somatic GHQ	0.859	0.133*	0.142**	0.071
Anxiety GHQ	0.890	0.148**	0.130*	0.134*
Dysfunction GHQ	0.747	0.053	0.053	0.033
Depression GHQ	0.844	0.075	0.067	0.066

*p < .05.

**p < .01.

Factor I was significantly and negatively related to Job satisfaction (*r =* -.300, *p* ≤ .01), the Empathy subscale of Standing in the Patient’s Shoes (*r =* -.142, *p* ≤ .01) and the Burnout dimension of Professional efficacy (*r =* -.226, *p* ≤ .01); and it was positively related to the Burnout dimension of Emotional exhaustion (*r =* .163, *p* ≤ .01) and the GHQ subscales of Somatic GHQ (*r =* .42, *p* ≤ .01) and Anxiety GHQ (*r =* .130, *p* ≤ .05). Factor II correlated significantly and negatively with Job Satisfaction (*r =* -.329, *p* ≤ .01), the Empathy subscales of Perspective taking (*r =* -.160, *p* ≤ .01) and Standing in the Patient’s Shoes (*r =* -.144, *p* ≤ .01), and the Burnout factor of Professional efficacy (*r =* -.176, *p* ≤ .01). It also correlated positively with the Burnout dimension Cynicism (*r =* .120, *p* ≤. 05) and with the Anxiety GHQ subscale (*r =* .134, *p* ≤ .05).

**Fig 1:**
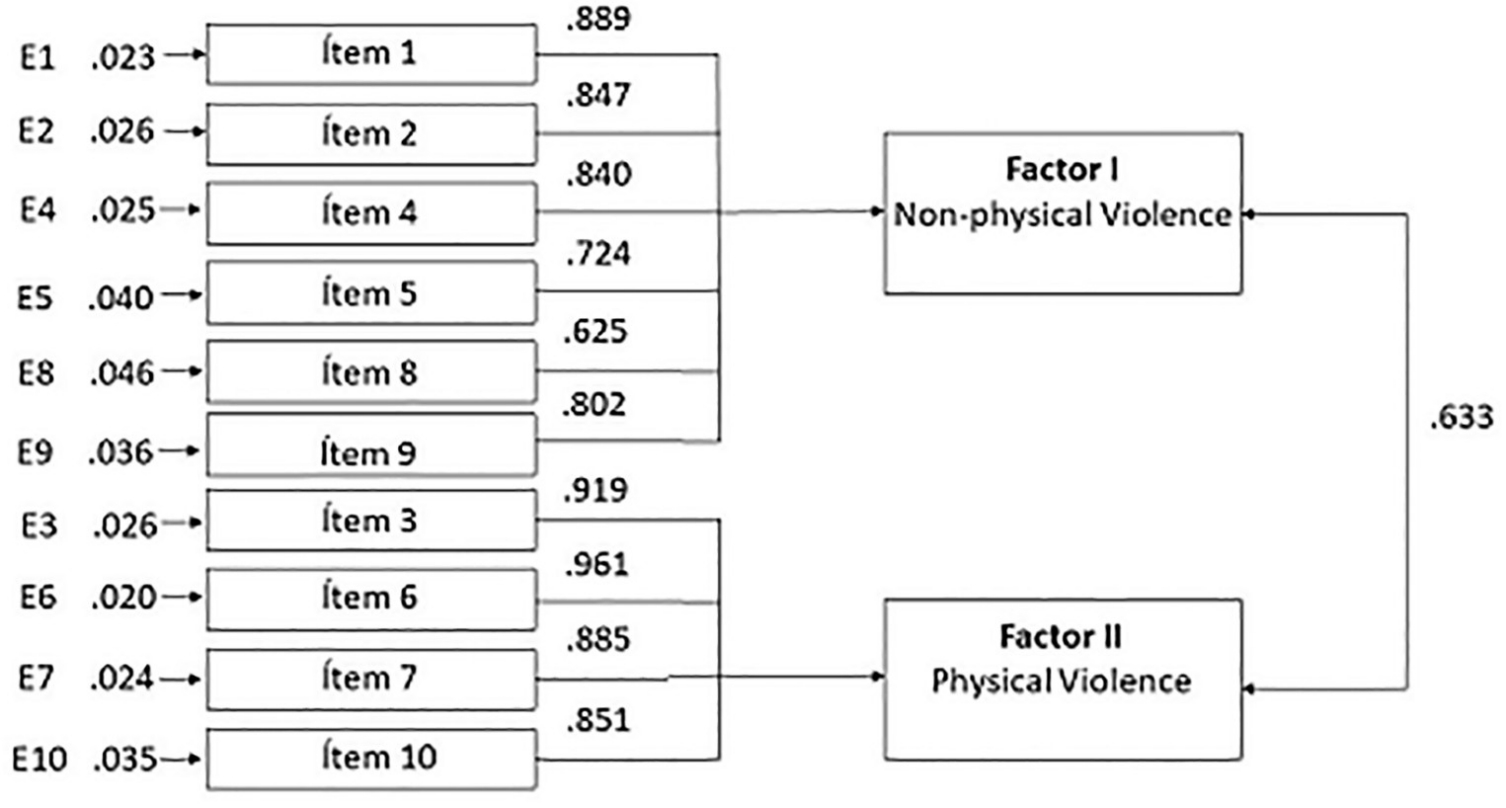
Analysis CFA.

The factor structure obtained was studied through CFA using the remaining 50% of the sample (*n* = 180, Fig 1). We found that the values of univariate skewness and univariate kurtosis of the items of Physical violence of the HABS-U-MH were not within the normal range (see Table 2), so we performed the CFA using the WLSMV (Mplus) estimation method, analyzing the following indices: CFI, RMSEA, and TLI.

The CFA supported the model of two correlated factors of the HABS-U-MH that had been identified by the EFA. The fit index values were adequate, CFI = 0.983, TLI = 0.978, RMSEA = 0.039, 95% CI [0.027, 0.051].

All the items presented loadings between 0.625 (Item 8, “Anger because of health care delay”) and .961 (Item 6, “Users have even shoved me, shook me, or spit at me”) (Fig 1). In the six items of Factor 1, the factor loadings ranged between 0.625 and 0.889, whereas in the four items of Factor 2, the factor loadings ranged between 0.851 and 0.961. Cronbach’s alpha coefficients were 0.877 for Factor I (Non-physical violence) and 0.841 for Factor II (Physical violence).

Analyzing the annual prevalence of user violence through the indicators employed, 92.1% of the professionals reported having been exposed to some type of violence: 90.7% to non-physical violence and 53.6% to physical violence. The most frequent (*at least monthly*) indicators of violence were, firstly, for patients to question the professional’s decisions in 35.8% of the participants, and secondly, the anger due to health care delay in 27.6%.

Table 5 shows the *ANOVA* that compares the perceived levels of violence in the four professional groups studied (psychiatrists, clinical psychologists, nurses, and non-medical personnel). The post hoc tests revealed statistically significant differences in the variables of Physical and Non-physical violence. Specifically, non-medical staff and nursing staff presented higher mean scores than the other professional groups, both in Non-physical (Tukey = 12.00, *p* < .001, ŋ^2^ = .088) and Physical violence (Tukey = 11.51, *p* < .001, ŋ^2^ = .109), whereas the psychology staff was notable for being the group with the lowest recorded rate, mostly of non-physical violence.

**Table 5:** Descriptive statistics and ANOVA of Non-physical and physical violence as a function of the professional group.

	Prof. group	*n*	*M*	*SD*	*F*	*p*	*df*	*Tukey*	ŋ^2^
**Non-physical violence**	A. Psychiatry	75	2.38	.98	12.00	.001	268	B-AC-CD	.088
	B. Psychology	45	1.85	.53					
	C. Nursing staff	102	2.74	1.37					
	D. Non-medical staff	49	3.25	1.45					
**Physical Violence**	A. Psychiatry	75	1.32	0.60	11.51	.001	268	BA-AD-DC	.109
	B. Psychology	45	1.06	0.15					
	C. Nursing staff	102	1.91	1.16					
	D. Non-medical staff	49	1.72	1.17					

Regarding perceived violence depending on the type of service (Table 6), the post hoc test showed that the Infant-Juvenile Unit and the Rehabilitation Unit reported the lowest rate of non-physical violence, whereas the Brief Inpatient Unit and the Medium-Stay Inpatient Unit reported the highest rates (Tukey = 6.05, *p* < .001, ŋ^2^ = .094). Regarding the indicators of physical violence, the Infant-Juvenile Unit, the Rehabilitation Unit, the Addictive Behaviors Unit, and Adult Unit obtained the lowest scores, whereas the Brief Inpatient Unit and the Medium-Stay Inpatient Unit were once again those reporting the highest levels of this type of violence (Tukey = 16.82, *p* < .001, ŋ^2^ = .224).

**Table 6:** Descriptive statistics and ANOVA of Non-physical and physical violence as a function of the type of service.

	Type of service	*n*	*M*	*SD*	*F*	p	df	Tukey	ŋ^2^
**Non-physical violence**	A. Adult Unit	112	243	1.16	6.05***	.001	296	BD-FE	.094
	B. Infant-Juvenile Unit	20	1.80	0.76					
	C. Addictive Behaviors Unit	36	2.42	1.14					
	D. Rehabilitation Unit	24	1.95	1.15					
	E. Brief Inpatient Unit	79	3.01	1.32					
	F. Medium-Stay Inpatient Unit	26	2.98	1.36					
**Physical Violence**	A. Adult Unit	112	1.25	0.60	16.82***	.001	296	BCAD- FE	.224
	B. Infant-Juvenile Unit	20	1.05	0.13					
	C. Addictive Behaviors Unit	36	1.18	0.45					
	D. Rehabilitation Unit	24	1.34	0.82					
	E. Brief Inpatient Unit	79	2.19	1.19					
	F. Medium Stay Inpatient Unit	26	2.07	2.07					

## Discussion and conclusions

For the development of this study, we applied the HABS-U to the area of Mental Health, obtaining a scale whose factors (Physical and Non-physical violence) are similar to those reported in previous studies. Also, when comparing our scale with the original scale (HABS-U) [24], developed within the hospital setting, and with the adaptation to the Primary Care population (HABS-U-PHC) [23], we found a set of common items that are maintained, preserving the initial factor structure. In each of the above adaptations, new items are added that reflect the characteristics of the different populations to which it was applied.

The internal consistency of the scale adapted to Mental Health has improved in comparison with the former two scales, as it obtained a higher alpha in Factor II, Physical violence (α = 0.84), versus the HABS-U of Wachsler, Ruiz-Hernández, Llor-Esteban, and Jimenez-Barbero [24], which had an alpha of 0.76, and the HABS-U-PHC of Ruiz Hernández *et al*. [23], which had an alpha of .68. This can be explained because violent behaviors involving physical aggression are more frequent in the Mental Health area, as is widely reflected in the existing literature [8, 10–13] and shown by the inclusion of one more item in the Physical violence subscale than in the two previous instruments.

Because of the diversity of physical and non-physical indicators and of the scales used to measure them, workplace violence prevalence varies considerably. In accordance with most of the studies [2, 9–11], we observed that non-physical violence indicators are more frequent than physical violence indicators among Mental Health professionals. The annual prevalence of non-physical violence was 90.7%, and for physical violence, it was 53.6%. In our study, the high exposure to physical violence (53.6% in the last month) was noteworthy, and it is usual to find these results in other investigations carried out in the context of Mental Health [9, 39, 40]. If we compare Mental Health with that of other health areas, we find that the rates are higher. Using the original version of the HABS-U, figures of 19.9% were obtained in hospital staff, and, more recently, Ruiz-Hernández *et al*. [23], in a study carried out with the same scale adapted to Primary Care, found values of 17.3% for physical violence. The high prevalence of physical assaults in Mental Health in comparison to other specialties can be explained by the close physical contact maintained with the patients, and by the special characteristics of the patients, because in this area, states of agitation and decompensation are frequent, sometimes requiring physical restraint [8, 9, 41].

In our study, non-medical staff and nursing staff are among the professionals most exposed to user violence, followed by medical personnel (Psychiatry) and, finally, the clinical psychology staff. The differences between the diverse Mental Health professional groups have been identified in other studies [9, 12, 42]. Magin, Joyce, Adams, Goode, and Cotter [43] reported that receptionists also are subject to considerably frequent workplace violence. The collective of non-medical professionals is acknowledged as being vulnerable to patients’ violent behaviors, given their position at the forefront of patient care and, therefore, they are the first to face users’ frustrations. Regarding the nursing staff, they have frequently been mentioned as the professionals most exposed to violence in Mental Health [16, 17, 20]. In this sense, it has been noted that 80% of the Mental Health nursing staff suffers violence, whereas in the rest of the clinical staff, this amount does not exceed 41% [10].

The group of clinical psychologists recorded the lowest rates, both of physical violence and non-physical violence. This may be due, on the one hand, to the fact that they are generally less exposed than the nursing staff and the non-medical staff, because they do not require close physical contact, like the nursing staff [7, 16, 40, 44], or bureaucratic procedures, like the administrative staff, which can provoke users’ impatience and discontent [43]. On the other hand, the psychologist’s work implies the need to establish a good therapeutic alliance as part of the intervention, for which an empathic attitude and unconditional acceptance of the patient are essential, which could explain the low frequency of aggressive behaviors [45].

Regarding the type of service within the Mental Health network, the facilities that reported the highest rates of violence, both physical and non-physical, were the Brief Inpatient Units and the Medium-Stay Inpatient Units. Our results are in line with the existing literature, as these units attend to users who require hospital admission, who are usually psychopathologically decompensated and, occasionally, their admittance is involuntary [15, 39]. A recent meta-analysis [17] revealed that approximately 20% of the patients admitted to Acute Psychiatric Units presented violent behavior.

From our work, we have drawn the conclusions set out below. On the one hand, the development of this study has allowed us to apply the HABS-U to the area of Mental Health. The obtained scale consists of 10 items distributed in two factors. It is short, easy to apply and interpret, and presents adequate psychometric properties and factor structure, so it can be concluded that it is useful to address user violence in Mental Health. On the other hand, just as in other health areas, we found that non-physical violence is more common than physical violence, but in Mental Health, physical violence reaches higher levels than in other services.

Regarding profession, nursing and non-medical staff are the most exposed to violent user behavior, and, within the Mental Health services, violence acquires relevant proportions in the inpatient psychiatric units, either in Acute Units or Medium-Stay Inpatient Units.

The present work has some limitations that are worthy of comment. Like all retrospective studies, it relies on participants’ recall, which may not be accurate. From the psychometric point of view, it would have been relevant to calculate test-retest reliability. As it was a large sample of professionals of the public health sector, the great health care demand faced by these workers on a daily basis makes it unfeasible to administer the protocol twice. As a strength of the study, we emphasize the high response rate (89.75%), in our opinion, generated by the field methodology used, unlike other similar studies [46].

This scale could be applied to better identify professionals exposed to user violence and to select appropriate individual and collective preventive measures, thereby reducing the psychological effects of violence. In addition, the use of the HABS-U-MH would make it possible to assess the effectiveness of intervention programs designed to minimize this problem.

The scale adapted to the Mental Health setting is included for dissemination as an assessment instrument (Annex 1), upon request to the authors.

## Supporting information

S1 Dataset (SAV)

S1 Appendix (PDF)
