# Supplementary material for: User violence in mental health services: Adaptation of an instrument. Healthcare-workers’ Aggressive Behavior Scale-Users-Mental Health Version (HABS-U-MH)
Source: PLoS One. 2019 Mar 4;14(3):e0212742. doi: 10.1371/journal.pone.0212742 (PMC6398850; doi:10.1371/journal.pone.0212742)
Supplement: S1 Appendix — (PDF) [file pone.0212742.s002.pdf]

Appendix 1 The Healthcare-workers' Aggressive Behavior Scale-Users-Mental Health (HABS-U-MH)

Healthcare-workers´ Aggressive Behavior Scale-Users-Mental Health

Below is a list of situations that may occur at your workplace. Please mark the frequency with which you have faced these situations. Please answer all the items using the following scale. **A** (N)ever **B** (A)nnually **C** (Q)uarterly **D** (M)onthly **E** (W)eekly **F** (D)aily

	**A**	**B**	**C**	**D**	**E**	**F**
1. Users question my decisions	N	A	Q	M	W	D
2. Users hold me exaggeratedly responsible for any trifle	N	A	Q	M	W	D
3. The users have even grasped me or touched me in a hostile manner	N	A	Q	M	W	D
4. Users accuse me unfairly of not fulfilling my obligations, committing errors or complications	N	A	Q	M	W	D
5. Users make ironic comments to me	N	A	Q	M	W	D
6. The users have even shoved me, shaken me, or spit at me	N	A	Q	M	W	D
7. Users show their anger at me by breaking doors, windows, walls. . .	N	A	Q	M	W	D
8. Users get angry with me because of assistential delay	N	A	Q	M	W	D
9. Users frown or give me contemptuous looks	N	A	Q	M	W	D
10. Users attacked me when I tried to avoid self-harm	N	A	Q	M	W	D

Cronbach α= 0.91; Mean= 1.94; Standard Deviation = 0.85

Factor I. Non-physical violence (Items 1, 2, 4, 5, 8, 9): Cronbach α = 0.890; Mean=2.55; Standard deviation =1.25

Factor II. Physical violence (Items 3, 6, 7, 10): Cronbach α = 0.893; Mean =1.55; Standard deviation =0.96
